# Combined Assessment of Preoperative Frailty and Sarcopenia Allows the Prediction of Overall Survival in Patients with Lung Cancer (NSCLC) and Surgically Treated Brain Metastasis

**DOI:** 10.3390/cancers13133353

**Published:** 2021-07-03

**Authors:** Inja Ilic, Anton Faron, Muriel Heimann, Anna-Laura Potthoff, Niklas Schäfer, Christian Bode, Valeri Borger, Lars Eichhorn, Frank A. Giordano, Erdem Güresir, Andreas H. Jacobs, Yon-Dschun Ko, Jennifer Landsberg, Felix Lehmann, Alexander Radbruch, Ulrich Herrlinger, Hartmut Vatter, Patrick Schuss, Matthias Schneider

**Affiliations:** 1 Department of Neurosurgery, University Hospital Bonn, 53127 Bonn, Germany; muriel.heimann@ukbonn.de (M.H.); anna-laura.potthoff@ukbonn.de (A.-L.P.); Valeri.Borger@ukbonn.de (V.B.); erdem.gueresir@ukbonn.de (E.G.); Hartmut.Vatter@ukbonn.de (H.V.); patrick.schuss@ukbonn.de (P.S.); matthias.schneider@ukbonn.de (M.S.); 2 Department of Radiology, University Hospital Bonn, 53127 Bonn, Germany; anton.faron@ukbonn.de; 3 Division of Clinical Neuro-Oncology, Department of Neurology, University Hospital Bonn, 53127 Bonn, Germany; niklas.schaefer@ukbonn.de (N.S.); Ulrich.Herrlinger@ukbonn.de (U.H.); 4 Department of Anesthesiology and Intensive Care Medicine, University Hospital Bonn, 53127 Bonn, Germany; Christian.Bode@ukbonn.de (C.B.); lars.eichhorn@ukbonn.de (L.E.); Felix.Lehmann@ukbonn.de (F.L.); 5 Department of Radiation Oncology, University Hospital Bonn, 53127 Bonn, Germany; frank.giordano@ukbonn.de; 6 Department of Geriatric Medicine and Neurology, Johanniter Hospital Bonn, 53113 Bonn, Germany; ahjacobs@uni-muenster.de; 7 Department of Oncology and Hematology, Johanniter Hospital Bonn, 53113 Bonn, Germany; yon-dschun.ko@bn.johanniter-kliniken.de; 8 Department of Dermatology and Allergy, University Hospital Bonn, 53127 Bonn, Germany; Jennifer.Landsberg@ukbonn.de; 9 Department of Neuroradiology, University Hospital Bonn, 53127 Bonn, Germany; Alexander.Radbruch@ukbonn.de

**Keywords:** brain metastases, non-small cell lung cancer, sarcopenia, frailty, overall survival, outcome

## Abstract

**Simple Summary:**

Patients with brain metastasis are at a severe stage of cancer, and brain surgery can prevent neurological morbidity. However, the success of brain surgery might require a patient’s physical integrity prior to the operation. In the present study, we asked whether a preoperative physical decline affects survival in patients with brain metastasis from lung cancer. In order to measure the physical condition, we used a commonly-known index—the so-called frailty index—and additionally measured the thickness of a particular masticatory muscle as muscle loss correlates to physical decline. We found that a decreased muscle thickness was accompanied by worsened survival for patients < 65 years and an increased frailty index correlated to worsened survival for patients ≥ 65 years. These results encourage to use of the frailty index and muscle thickness as easily available parameters in order to more sufficiently estimate individual treatment success in patients with metastatic lung cancer.

**Abstract:**

Neurosurgical resection represents an important therapeutic pillar in patients with brain metastasis (BM). Such extended treatment modalities require preoperative assessment of patients’ physical status to estimate individual treatment success. The aim of the present study was to analyze the predictive value of frailty and sarcopenia as assessment tools for physiological integrity in patients with non-small cell lung cancer (NSCLC) who had undergone surgery for BM. Between 2013 and 2018, 141 patients were surgically treated for BM from NSCLC at the authors’ institution. The preoperative physical condition was assessed by the temporal muscle thickness (TMT) as a surrogate parameter for sarcopenia and the modified frailty index (mFI). For the ≥65 aged group, median overall survival (mOS) significantly differed between patients classified as ‘frail’ (mFI ≥ 0.27) and ‘least and moderately frail’ (mFI < 0.27) (15 months versus 11 months (*p* = 0.02)). Sarcopenia revealed significant differences in mOS for the <65 aged group (10 versus 18 months for patients with and without sarcopenia (*p* = 0.036)). The present study confirms a predictive value of preoperative frailty and sarcopenia with respect to OS in patients with NSCLC and surgically treated BM. A combined assessment of mFI and TMT allows the prediction of OS across all age groups.

## 1. Introduction

Non-small cell lung cancer (NSCLC) is one of the most common primary tumor types in patients suffering from brain metastases (BM) [1]. Approximately 10% of patients have existing BM at initial diagnosis, whereas roughly 40% of patients develop BM during the treatment course of their underlying malignancy [1,2]. Neurosurgical resection of BM represents an important part of multimodal oncological therapy with regard to cytoreduction and histological insights [3]. Genetic profiling has become increasingly important in this context [4]. However, such extended multimodal treatment modalities require a valid assessment of the extent of a patient’s functional and physical integrity. Along these lines, the frailty index as a preoperative estimation of physical resources has been established as a predictor for treatment success in several diseases [5,6,7]. There is increasing evidence that the physician’s assessment of frailty needs to be supplemented by additional, objectifiable indicators to approximate the preferred optimized full-scope assessment. Notably, it is well known that in cancer patients, lean muscle mass serves as a predictor of impaired mobility and increased mortality [8,9]. In particular, tumor-associated cachexia and sarcopenia have been demonstrated to be associated with poor overall survival (OS) [10]. In order to determine sarcopenia preoperatively, temporal muscle thickness (TMT) has previously been identified as a predictor of OS in patients suffering from BM [11]. With regard to TMT to significantly correlate to a patient’s skeletal muscle mass, TMT has been widely used as a surrogate parameter for sarcopenia [12]. There are several methods to determine muscle quantity/quality for sarcopenia assessment. In this context, MRI and CT measurements are the gold standard for the acquisition of muscle quantity/quality [13,14]. A high correlation between lumbar skeletal muscles and TMT in patients with BM has been found [15].

In order to allow a more holistic preoperative estimation of the physical resources, the aim of the present study was to determine the potential impact of both parameters, sarcopenia and frailty, in terms of their preoperative predictive value on OS in patients with NSCLC and surgically treated BM.

## 2. Materials and Methods

### 2.1. Patients

Between 2013 and 2018, 388 patients with newly diagnosed BM were surgically treated at our institution. Among these, a total of 154 patients with histopathologically proven NSCLC were identified and included in further analysis. Patients in whom preoperative magnetic resonance imaging (MRI) scans were not available by the time of the analysis were excluded due to the inability to obtain the intended measurements. Data including patient characteristics, radiological features, neurological status on admission and during the treatment course were collected, pseudonymized, and entered into a computerized database (SPSS, version 27, IBM Corp., Armonk, NY, USA). In addition, we analyzed molecular profiling for epidermal growth factor receptor (EGFR) mutation, anaplastic lymphoma kinase (ALK) translocation, and anti-programmed cell death 1 ligand 1 (PD-L1) expression, if detected. Karnofsky’s performance status (KPS) was used to assess the neurological functional status of the patients analyzed. Patients were divided into two groups based on the KPS according to standard practice: KPS ≥ 70 versus (vs.) KPS < 70.

For a more detailed description of the possible preoperative comorbidity burden of the patients, the age-adjusted Charlson comorbidity index (CCI), as well as the American Society of Anesthesiologists (ASA) score, was applied. Individual treatment decisions were based on an interdisciplinary, patient-focused consensus during weekly institutional interdisciplinary tumor board meetings at our neuro-oncology specialty center [16,17].

### 2.2. Modified Frailty Index

To additionally analyze the overall level of fitness/frailty, the modified frailty index (mFI) was used as described previously [18]. In detail, the mFI consists of 11 items to be determined preoperatively. For index calculation, each item was allocated the same number of points and thus the same weight. The mFI was then computed for each individual patient by dividing the patient-specific total score by the sum of all items [19]. Although the mFI was not designed as a dichotomized variable, patients were assigned to three groups based on previous experience according to their mFI: "least frail" (mFI 0.00–0.08), “moderately frail” (mFI 0.09–0.26), and “frailest” (mFI ≥ 0.27) [5].

### 2.3. Temporal Muscle Thickness Measurement

**Figure 1:**
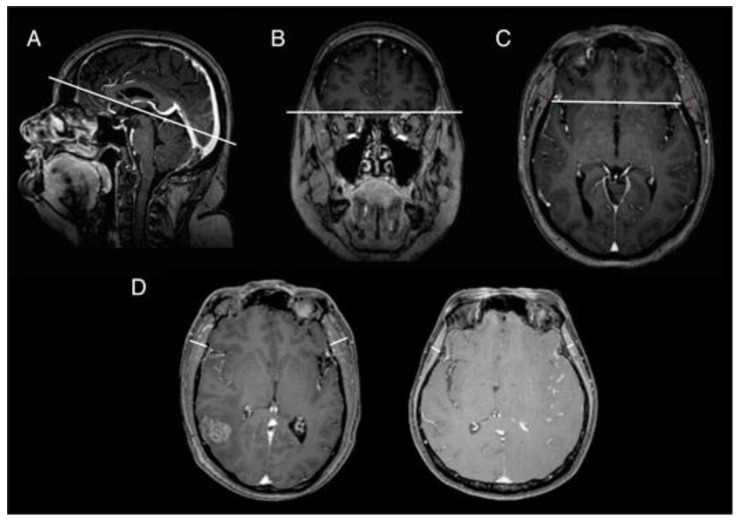
Example of TMT measurements using standard isotropic contrast-enhanced T1-weighted cranial MRI (**A**–**C**) demonstrate the MRI alignment for TMT acquisition. (**D**) A 57-year-old male without sarcopenia (left), a 48-year-old female with sarcopenia (right). MRI, magnetic resonance imaging; TMT, temporal muscle thickness.

Preoperative cranial 3D MRI scans were performed in all patients. Single-slice measurements from standard preoperative MRI scans were utilized for TMT calculation. Measurements were performed perpendicular to the long axis of the temporalis muscle as previously described [10,19], using a standard isotropic contrast-enhanced T1-weighted sequence. For this purpose, the transversal plane was aligned parallel to the anterior–posterior-commissure line (Figure 1A), using the orbital roof (Figure 1B) and Sylvian fissure (Figure 1C) as anatomical landmarks for cranio–caudal and anterior–posterior orientation, respectively. Figure 1D illustrates an example for a patient without and with sarcopenia (Figure 1D). All TMT assessments were conducted by a board-certified radiologist (AF). The radiologist was blinded to all clinical patient characteristics, treatment course, and OS. TMT measurements were evaluated separately on each side. After summation of both sides, the mean TMT per patient was calculated.

### 2.4. Statistics

Data analyses were performed using the computer software package SPSS (version 25, IBM Corp., Armonk, NY, USA). Categorical variables were analyzed in contingency tables using Fisher’s exact test. The Mann–Whitney *U*-test was chosen to compare continuous variables as the data were mostly not normally distributed. Results with *p* < 0.05 were considered statistically significant. OS was analyzed by the Kaplan–Meier method using the Gehan–Breslow–Wilcoxon test. A multivariate Cox regression model was applied to determine independent variables for worsened OS in cancer patients that had undergone surgery for BM from NSCLC.

## 3. Results

### 3.1. Patient Characteristics

In total, charts of 141 patients with NSCLC and BM requiring surgery were subjected to further analyses (Table 1). The median age was 64 years (IQR 58–70). Forty-nine percent of included patients were female (69 vs. 72). In 50 patients (36%), multiple BM were present in addition to the BM requiring surgery at the time of surgery. In 48 patients (34%), the BM requiring surgery was located in the posterior fossa. For further information regarding molecular subtype classification of the underlying NSCLC disease, see also Figure S1.

### 3.2. Influence of Frailty on Overall Survival

**Figure 2:**
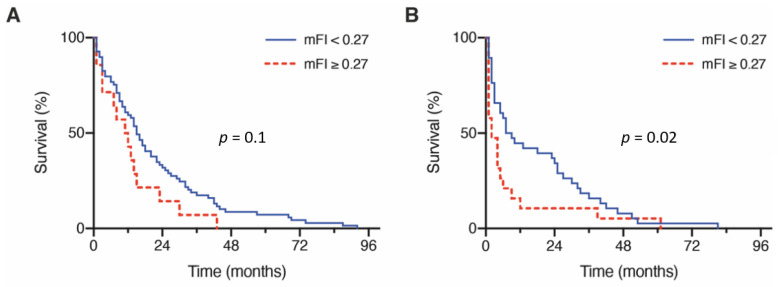
Patient frailty correlations to OS rates stratified for (**A**) patients aged < 65 y and (**B**) patients ≥ 65 y. y, years.

Within the preoperative assessment regarding frailty status, patients with BM from lung cancer had a median mFI of 0.09. In-depth incidences of conditions reflected by the mFI are provided in Table 2. Analysis of mortality rates revealed a significantly higher likelihood of death with a higher mFI (*p* = 0.002; Figure S1A). After stratification for mFI, patients with NSCLC and BM classified as least frail and moderately frail (mFI < 0.27) achieved a mOS of 14 months (95% CI 9.4–18.6), whereas the frailest patients (mFI ≥ 0.27) achieved a mOS of 4 months (95% CI 1.1–6.9; *p* = 0.001, Figure S1B). After subdividing the studied cohort of patients by age, the mOS for patients < 65 years with an mFI < 0.27 (*n* = 63) was 15 months (95% CI 11.7–18.3), compared to 11 months for patients < 65 years with an mFI ≥ 0.27 (*n* = 14) (95% CI 4.7–17.3; *p* = 0.1, Figure 2A). For the group of patients with BM from lung cancer requiring surgery aged ≥ 65 years, the corresponding values for mOS were 7 months (*n* = 44) (95% CI 2.2–11.8; mFI < 0.27) versus 2 months (*n* = 209 ) (95% CI 0.0–4.6; mFI ≥ 0.27; *p* = 0.02, Figure 2B).

### 3.3. Influence of Sarcopenia on Overall Survival

**Figure 3:**
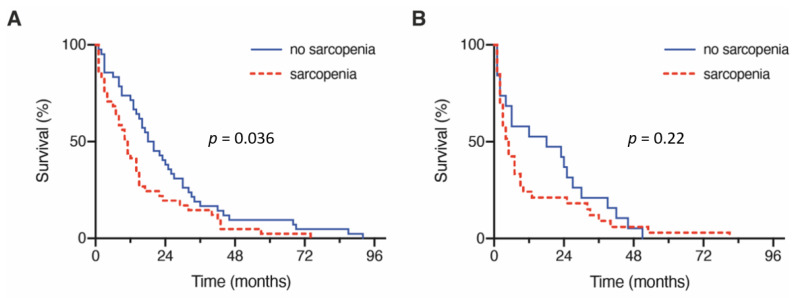
Patient sarcopenia correlations to OS rates stratified for (**A**) patients aged < 65 y and (**B**) patients ≥ 65 y. y, years.

The preoperatively measured TMT values in patients with lung cancer and BM requiring surgery resulted in an overall median TMT value of 10.5 mm (IQR 7.8–13). Dividing the patient cohort according to the defined cutoff into a group without preoperative sarcopenia (median TMT ≥ 11 mm) and a group with preoperative sarcopenia (median TMT < 11 mm) resulted in group sizes of 62 patients (44%, no sarcopenia) and 79 patients (56%, sarcopenia), respectively. Analysis of mortality rates revealed a significantly higher likelihood of death dependent on the presence of sarcopenia (one-year mortality rate without sarcopenia: 34%; with sarcopenia: 68%; Figure S2A). The mOS in patients with preoperatively identifiable sarcopenia was 7 months (95% CI 3.8–10.2) compared with 18 months in patients without preoperative sarcopenia (95% CI 12.0–24.0; *p* = 0.006; Figure S2B). After subdividing the studied cohort of patients by age, the mOS for patients < 65 years without sarcopenia (*n* = 38) was 18 months (95% CI 11.6–24.4), compared to 10 months for patients < 65 years with sarcopenia (*n* = 39) (95% CI 6.8–13.2; *p* = 0.036, Figure 3A). However, for patients with BM from lung cancer requiring surgery aged ≥ 65 years, the corresponding values for mOS were 18 months (*n* = 24) (95% CI 0.0–42.1; without sarcopenia) versus 4 months (*n* = 40) (95% CI 2.0–6.0; with sarcopenia; *p* = 0.22, Figure 3B).

### 3.4. Multivariate Analysis

**Figure 4:**
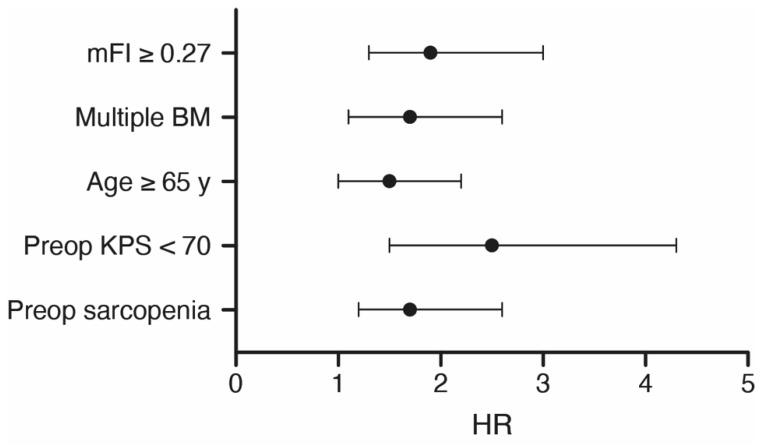
Results from the multivariate analysis. BM, brain metastasis; HR, hazard ratio; KPS, Karnofsky’s performance status; mFI, mean frailty index; y, years.

Additionally, multivariate survival analysis was performed to identify independent preoperative predictors of OS in patients with lung cancer and surgically treated BM. The multivariate Cox regression model revealed the preoperative variables “preoperative sarcopenia (TMT < 11 mm)” (*p* = 0.004, HR 1.7, 95% CI 1.2–2.6), “frailest admission status (mFI ≥ 0.27)” (*p* = 0.002, HR 1.9, 95% CI 1.3–3.0), “preoperative KPS < 70%” (*p* = 0.001, HR 2.5, 95% CI 1.5–4.3), “age ≥ 65 years” (*p* = 0.03, HR 1.5, 95% CI 1.0–2.2), and “multiple BM” (*p* = 0.01, HR 1.7, 95% CI 1.1–2.6) as significant and independent predictors of poor OS in the present patient cohort (Figure 4). This section may be divided by subheadings. It should provide a concise and precise description of the experimental results, their interpretation, as well as the experimental conclusions that can be drawn.

## 4. Discussion

The present study describes the influence of physical resources on OS in patients with NSCLC and BM requiring surgery. Hereby, it is evident that both used measurement techniques for the assessment of physical resources might have a high preoperative predictive value concerning OS of these affected patients.

The physical and clinical condition of a cancer patient is key for choosing adequate treatment modalities and intensities. However, the assessment of such is inhomogeneously performed and may vary from treatment center to treatment center. It thus remains challenging to predict which factors that, alone or in sum, constituting the ‘global’ condition, are most indicative for a benefit from a corresponding treatment (or intensity) [18].

The presence of BM resembles an advanced stage of cancer, requiring optimal dissection of factors that mount to a ‘global’ performance index (such as the KPI). Here, specifically for elderly patients, options to approach the impression of physical resources (frailty) of a patient by means of clinical scores have been proven useful for this purpose [5,6]. Both coding-based scores and more subjective scales can be used to determine the frailty of a patient [19]. In clinical practice, more subjective scores, such as the Clinical Frailty Scale, appear somewhat closer to the real world [6,20]. For (retrospective) data processing in the context of clinical studies, coding-based solutions, such as the modified Frailty Index, seem to be more resistant to confounding. However, all frailty scores ultimately share the subjective perception of the treating physician.

In the search for more objectifiable measurement techniques for determining (preoperative) frailty, the detection of sarcopenia is often touted [12,21,22]. Sarcopenia in cancer patients is interpreted as an embodiment of a multifactorial phenomenon. In addition to the consumptive effects of the malignancy itself and subsequent treatments, which are expressed in the form of inflammatory and/or catabolic changes, the altered mobility and this physical endurance of the patient also contribute to this process [23,24]. Furthermore, it has already been verified that sarcopenia in NSCLC is a disadvantageous prognostic factor [25].

Muscle quantity/quality is only one parameter of the three parameters defining sarcopenia [13]. The measuring of grip strength to evaluate the muscle strength was not assessed. We also did not measure the physical performance by using Short Physical Performance Battery (SPPB) or the Timed-Up and Go-test (TUG). However, the mFI, as well as the KPS, include the functional health status prior to surgery, which might also serve as a physical performance tool.

By means of an objectifiable measuring method, sarcopenia is detected, for example, by the thickness of the temporal muscle as a proxy for the overall constitution of the skeletal muscle system [26]. Various research groups have already demonstrated the predictive value of sarcopenia/TMT in different diseases, such as glioblastoma, aneurysmal subarachnoid hemorrhage, or CNS lymphoma [12,27]. As evidenced by the multivariate analysis of the present study, there is a strong and independent influence of preoperative determined TMT as an expression of sarcopenia on the OS patients with BM of NSCLC requiring surgery. A similar relationship has previously been demonstrated by Furtner et al. in patients with newly diagnosed BM [11,28].

Interestingly, the age-dependent analysis of the data of the present study, however, revealed an apparent deficiency of the frailty evaluation using TMT/sarcopenia in elderly patients (≥65 years). Here, OS ceased to be significantly different between older patients with and without preoperatively defined sarcopenia (*p* = 0.22). This shortcoming seems to be overcome by frailty determination based on the mFI. The mFI also demonstrated a strong and independent predictive value with respect to OS in patients with NSCLC and surgically treated BM, according to the present multivariate analysis. However, in the age-dependent analysis, the mFI, inversely to the sarcopenia measure, henceforth revealed limitations in its predictive value in younger patients (<65 years). Here, the mFI demonstrated no significant survival difference between younger patients classified as least frail/moderately frail and those classified as frailest (*p* = 0.1).

In the context of cancer treatment, individualized immunotherapy is gaining relevance [4]. One of the determining aspects is the molecular profile of the tumor. Nevertheless, several studies have revealed a reduced OS in patients with sarcopenia despite treatment with immune checkpoint inhibitors [29,30]. In addition, positive PD-L1 expression was reported more frequently in patients with primary brain metastases [31]. In the present patient population, the analysis of PD-L1 expression could only be performed in 22%. Among these, positive PD-L1 expression was detected in 16 patients (11%). With regard to the retro-perspective data collection within the present series, which dates back to 2013, consistent detection of this prognostic molecular marker was not possible.

The results of our study indicate an appropriate predictive value of preoperative frailty assessment of patients with NSCLC and BM for OS. Nevertheless, the age-adjusted interpretation of the present data highlights the importance of not relying on the validity of a single scale and/or measurement in the complex assessment of these patients. Rather to the contrary, for patients of this degree of complexity, the clinical reality might be better reflected by looking at few but well-defined parameters, as studied here. The frailty of a patient is certainly a useful and established approach to guide an individualized, patient-centered medical decision in a collaborative discussion/decision-making process with the patient and/or relatives. However, a more comprehensive analysis, including clinical (here: mFI), as well as neuroimaging (here: sarcopenia/TMT) parameters in patients with NSCLC and BM seems to be necessary to overcome the respective limitations. Subsequent prospective rather multicenter studies will be needed to more sufficiently evaluate the influence of frailty and sarcopenia on OS in patients with BM, especially with regard to the molecular–genetic subclasses of NSCLC.

### Limitations

Retrospective data collection and interpretation is the most obvious limitation of the present study. In addition to data acquisition from a single center, the use of mFI and TMT as measurement tools for frailty does not encompass all established factors of frailty. In addition, the focus on lung cancer as the cause of BM and the need for surgical intervention for that same BM pose the risk of selection bias. Nevertheless, this intentional selection also avoids extensive heterogeneity among the analyzed patients with BM. With regard to the retrospective data collection passing back into the year 2013, a consistent collection of known molecular prognostic markers (PD-L1, EGFR-mutation, ALK-, and ROS1-rearrangement) was not possible and consideration for the multivariate Cox regression analysis, therefore, was not sufficient. Despite the shortcomings mentioned above, the standardized collection of data, particularly by a radiologist blinded to all clinical parameters at the time of TMT measurements, improved the conclusiveness of the data compiled here. Nevertheless, further clinical research is necessary and desirable to clarify the presumed benefit of combined consideration of mFI and TMT with regard to frailty.

## 5. Conclusions

The present study confirms a substantial independent predictive value of frailty and sarcopenia as preoperatively collectable variables with respect to OS in patients with NSCLC and surgically treated BM. The age-adjusted analysis yielded an increased degree of reliability across all age groups when combining frailty and sarcopenia as highly standardized assessment tools for physical integrity.

## Figures and Tables

**Table 1:** Patient Characteristics.

Patient Characteristics	Patients with NSCLC and BM Requiring Surgery (*n* = 141)
median age (y, IQR)	64 (58–70)
female sex	69 (49%)
preoperative KPS ≥ 70%	120 (85%)
preoperative ASA ≥ 3	81 (57%)
age-adjusted CCI > 10	74 (53%)
median TMT (mm, IQR)	10.5 (7.8–13)
modified mFI	
least frail (0.00–0.08)	31 (22%)
moderately frail (0.09–0.26)	76 (54%)
frailest (≥0.27)	34 (24%)
location posterior fossa	48 (34%)
multiple BM	50 (36%)
anticoagulation medication prior surgery	37 (26%)
overall survival (mo)	11 (95% CI 7.6–14.4)

NSCLC, non-small cell lung cancer; BM, brain metastasis; y, years; IQR = interquartile range; KPS, Karnofsky’s performance status; ASA, American Society of Anesthesiologists; CCI, Charlson Comorbidity index; TMT, temporal muscle thickness; mFI, modified frailty index; mo, months; CI, confidence interval.

**Table 2:** Frequency of Patient Frailty According to the Modified Frailty Index.

Index Weight	Index Weight	Frequency % (*n*)
1	Functional health status prior surgery (only dependent)	43 (61)
1	History of diabetes mellitus	12 (17)
1	History of severe COPD/current pneumonia	27 (38)
1	Congestive heart failure	4 (6)
1	History of myocardial infarction	1 (2)
1	Previous percutaneous coronary intervention; previous cardiac surgery; history of angina	10 (14)
1	Hypertension requiring medication	45 (63)
1	Impaired sensorium	6 (8)
1	History of transient ischemic attack	0.7 (1)
1	Cerebrovascular accident/stroke with neurologic deficit	6 (8)
1	History of revascularization for peripheral vascular disease	9 (13)

COPD, chronic obstructive pulmonary disease.

## Data Availability

The authors confirm that the data supporting the findings of this study are available within the article and its Supplementary Materials.

## Supplementary Materials

The following are available online at https://www.mdpi.com/article/10.3390/cancers13133353/s1, Figure S1: (A) 30 days and one-year mortality rate in patients according to the mFI cutoff value as indicated. (B) Kaplan–Meier curves for OS of patients according to the mFI cutoff value as indicated, Figure S2: (A) 30 days and one-year mortality rate in patients according to the presence or absence of sarcopenia. (B) Kaplan–Meier curves for OS of patients according to the presence or absence of sarcopenia. Table S1: Overview of the distribution of known molecular biomarkers in the present cohort of NSCLC patients.
