# Supplementary material for: Combined Assessment of Preoperative Frailty and Sarcopenia Allows the Prediction of Overall Survival in Patients with Lung Cancer (NSCLC) and Surgically Treated Brain Metastasis
Source: Cancers (Basel). 2021 Jul 3;13(13):3353. doi: 10.3390/cancers13133353 (PMC8267959; doi:10.3390/cancers13133353)
Supplement: Supplementary file 1 [file cancers-13-03353-s001.zip › cancers-1270462-Table S1.pdf]

Article

Combined Assessment of Preoperative Frailty and Sarcopenia Allows the Prediction of Overall Survival in Patients with Lung Cancer (NSCLC) and Surgically Treated Brain Metastasis

Inja Ilic [1,†,*], Anton Faron, [2,†], Muriel Heimann [1], Anna-Laura Potthoff [1], Niklas Schäfer [3], Christian Bode [4], Valeri Borger [1], Lars Eichhorn [4], Frank A. Giordano [5], Erdem Güresir [1], Andreas H. Jacobs [6], Yon-Dschun Ko [7], Jennifer Landsberg [8], Felix Lehmann [4], Alexander Radbruch [9], Ulrich Herrlinger [3], Hartmut Vatter [1], Patrick Schuss, [1,‡] and Matthias Schneider [1,‡]

Table S1. Overview of the distribution of known molecular biomarkers in the present cohort of NSCLC patients*.

	**Expression**		
	**n.a.**	**+**	**–**
PD-L1	110 (78)	16 (11)	15 (11)
EGFR-mutant	93 (66)	4 (3)	44 (31)
ALK-rearranged	92 (65)	1 (1)	48 (34)
ROS1-rearranged	121 (86)	1 (1)	19 (13)

*** Values represent number of patients unless otherwise indicated (%).** ALK, anaplastic lymphoma kinase; EGFR, epidermal growth factor receptor; n.a., not applicable; NSCLC, non-small cell lung carcinoma; PD-L1, anti-programmed cell death 1 ligand 1.
