# Supplementary figures and images for: Combined Assessment of Preoperative Frailty and Sarcopenia Allows the Prediction of Overall Survival in Patients with Lung Cancer (NSCLC) and Surgically Treated Brain Metastasis
Source: Cancers (Basel). 2021 Jul 3;13(13):3353. doi: 10.3390/cancers13133353 (PMC8267959; doi:10.3390/cancers13133353)

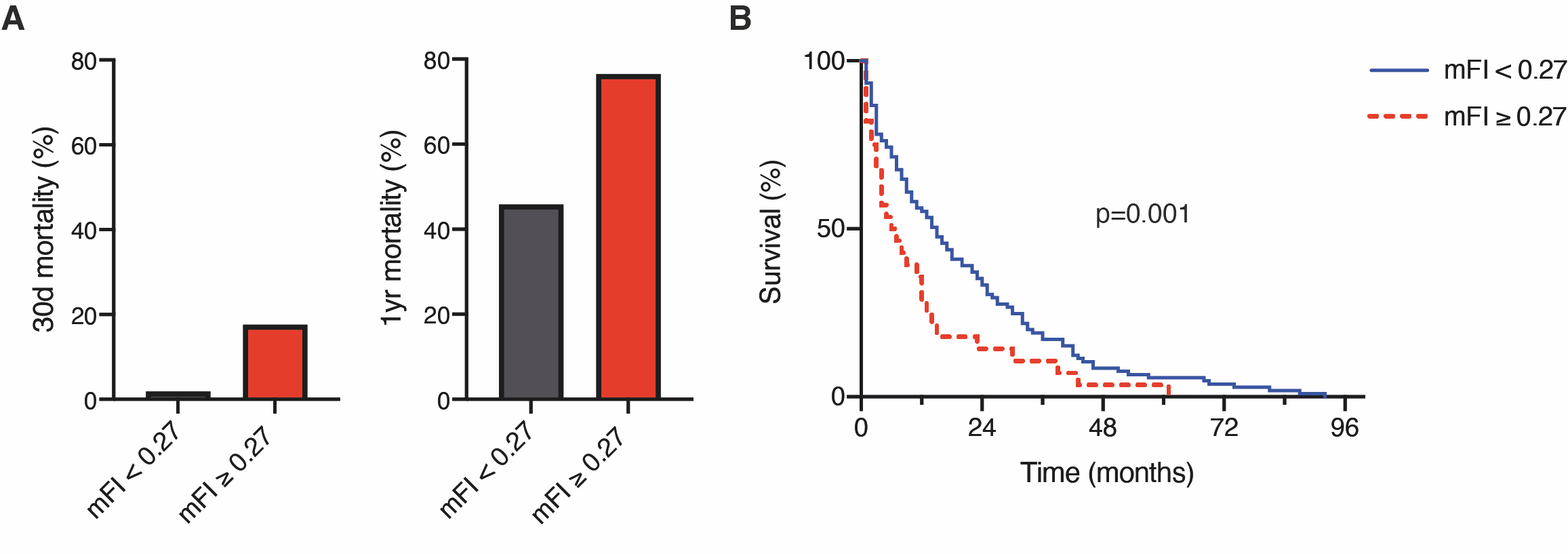

Supplement: Supplementary file 1 [file cancers-13-03353-s001.zip › Supplementary Figure S1.tiff]

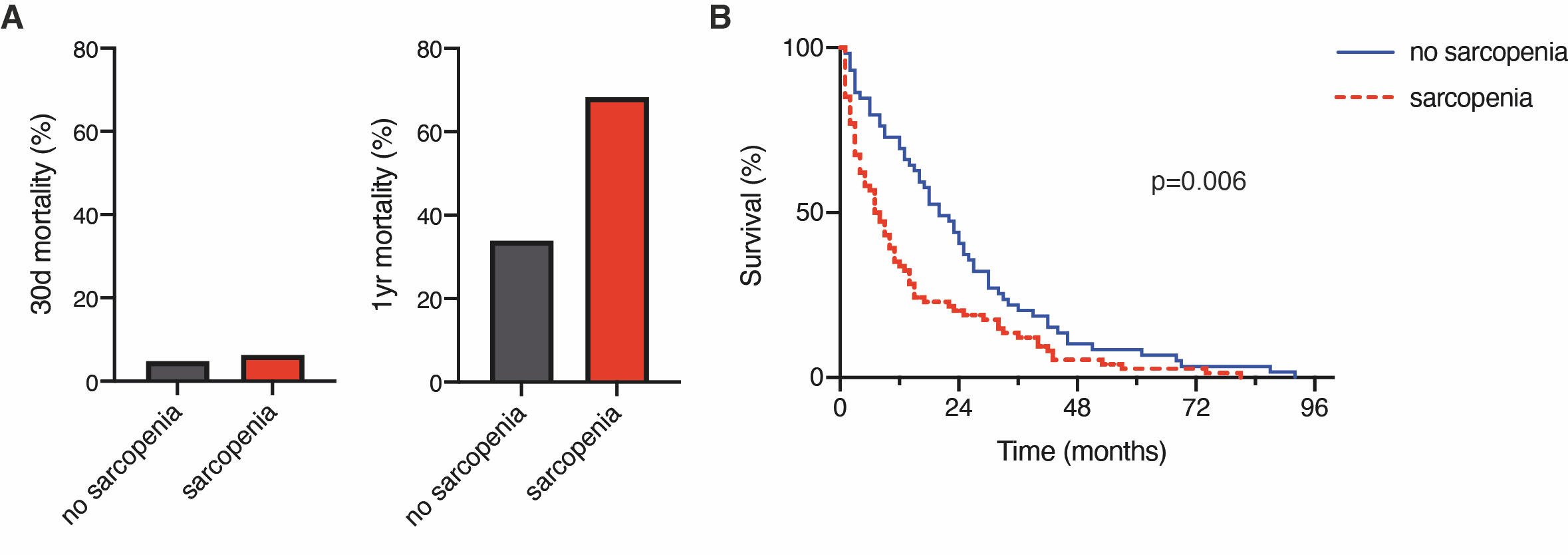

Supplement: Supplementary file 1 [file cancers-13-03353-s001.zip › Supplementary Figure S2.tiff]
